# Physical Fighting among School-Attending Adolescents in Pakistan: Associated Factors and Contextual Influences

**DOI:** 10.3390/ijerph16245039

**Published:** 2019-12-11

**Authors:** Masood Ali Shaikh, Anne Abio, Karen L. Celedonia, Michael Lowery Wilson

**Affiliations:** 1 Injury Epidemiology and Prevention Research Group, Turku Brain Injury Centre, Division of Clinical Neurosciences, Turku University Hospital and University of Turku, 20521 Turku, Finland; masoodalishaikh@gmail.com (M.A.S.); anne.p.abio@utu.fi (A.A.); karen.k.celedonia@utu.fi (K.L.C.); 2 Injury Epidemiology and Prevention Research Unit, Heidelberg Institute of Global Health, University of Heidelberg, 69120 Heidelberg, Germany

**Keywords:** interpersonal violence, school health, epidemiology, global health, mental health

## Abstract

Background: Adolescent physical fighting is a problem of public health importance, with varied consequences in the form of school absenteeism, injury, and, in some cases, death. Although research on risk and protective factors exists, most has been conducted in high-income countries. Methods: The 2009 Pakistan Global School-based Health Survey (GSHS) data were used. Logistic regression models were used to determine the associations. Five independent variables were investigated at the individual level (anxiety, suicide planning, truancy, physical activity, and bullying victimization) and four independent variables at the social level (presence of supportive parental figures, presence of helpful peers, extent of social network, and food insecurity). Results: Among adolescents in this study (N = 5177), 20% reported being involved in two or more physical fights, most of whom were males (79.9%). The factors associated with physical fighting were: being male (OR = 2.78); bullying victimization (OR = 3.14); truancy (OR = 1.63), loneliness (OR = 1.44); and suicidality, as evidenced by having a suicide plan (OR = 1.75). Having few close friends (0–2) as opposed to more (>3) was found to be protective against engaging in physical fighting. Conclusion: Risk factors for physical fighting among adolescents in South Asia seem to corroborate with previously-identified risk factors using samples in high-income countries, while protective factors seemed to differ. More research needs to be conducted to understand why certain factors do not have the same protective effect among South Asian adolescents. Aim: The aim of this study was to examine demographic and contextual factors associated with physical fighting among a nationally representative sample in a rapidly developing South Asian context.

## 1. Introduction

Physical fighting among adolescents in school settings is a problem of public health importance worldwide [1,2]. When one or more adolescents decide to engage in a physical fight, it can potentially lead to injuries requiring medical attention, or result in more serious outcomes, including death [3,4]. According to the US Centers for Disease Control (CDC), youth violence is defined as when young people aged 10–24 years deliberately use physical force or power to threaten and/or harm others. Globally, in 2013, the mortality due to youth violence among adolescents aged 15–19 years was ranked fourth at 5.5%; and more specifically among males, it was ranked second at 7.8% [5,6].

In the Middle East and Africa, at least half of studied adolescents have reported being involved in at least one physical fight during the year prior to being surveyed [4,7]. Among studies conducted in Asia, the prevalence of physical fighting among school attending adolescents ranged from 14% to 38% [8,9,10,11]. A study conducted among victims of intentional injuries in Pakistan found approximately 14% of victims were adolescents aged 11–20 years [12].

Several sociodemographic factors have been found to be related with violent behavior among adolescents. These include: substance misuse and abuse, behavioral problems, emotional distress, inadequate parental supervision, and bullying victimization [2,3,4,6,7,13,14]. Being male has often been found to be associated with youth violence in among several studies making use of nationally representative data [3,4,6,15,16]. Although most studies report an over-representation of males in physical fights [2,4,17,18], the gap between males and females involved in physical fighting appears to be narrowing over time [17]. Bullying victimization is also associated with youth violence as victims may defend themselves from perpetrators and in some cases individuals have assumed both the role of victim and perpetrator [19]. In light of this, adolescents involved in physical fights may do so as a result of having experienced bullying. School absence has also been found to be associated with youth violence [2,4,6] with some students who do so report being fearful of harassment in school according to the CDC. On the other hand, truancy has been linked to behaviors such as substance use, smoking, and alcoholism, which may affect performance in school but are also associated with increased violence [6,7,20]. Youth violence is a predictor of depressive symptoms, for example suicide ideation and planning, and adolescents involved in violence tend to have suicidal thoughts [21]. Longitudinal studies conducted in the US have found violence to be a predictor of suicidality [21,22]. In some studies, sedentary behavior has been associated with physical fighting [4,16]. Physical activity has been found to be protective from youth violence in some studies [16] but showed no association in others [4]. Reduced rates of violent behavior among adolescents have largely been found within settings in which parental guidance is optimal, among youth who have helpful supportive peers and within positive school environments [2,4]. During the formative years of adolescence where peer-acceptance, rapid transitions in physiological and psychological development occurs, various forms of aggressive behavior could possibly manifest [23,24]. Adolescence is a period of self discovery and exploration during which adolescents are likely to engage in certain behaviors and spend more time with peers, and adequate parental support has been found to protect them from negative behavior [25,26]. However the amount of time they spend with peers versus family tends to determine the amount of influence on their respective behavior [25]. A study conducted among adolescents in Pakistan found physical aggression to be associated with a permissive parenting style, which was described as parents who overlooked their children’s negative behavior [27]. Thus, parents and/or guardians as well as peers play a significant role in shaping the conduct of adolescents. Furthermore, the availability of helpful and supportive peers is also protective from youth violence [4]. Adolescents will tend to select friends based on similarities in character, behavior, or admiration [25]. On the contrary, peers who engage in negative behavior may influence one another to engage in problematic activities [25]. Higher socioeconomic status has been found to be protective of youth violence [3]. In this regard, schools considered to be high quality or with a positive environment have also been found to be protective [2,16].

Physical fighting has also been associated with injuries, threats, intimidation, fear and vulnerability, absenteeism from school, dropping out of school, medical costs, and disruption of studies [2,3,13,20]. One study on the trend of physical fighting over time found that it has declined in the US and most European countries except Greece, Latvia, and Ukraine, specifically from 2002 to 2010 [3]. The majority of the research on adolescent and youth violence has been done in high-income countries, and to a less extent among the middle- and low-income countries [2,4]. The aim of this study was to examine demographic and contextual factors associated with physical fighting among a nationally representative sample in a rapidly developing South Asian context. Findings from the present study add to the growing body of epidemiologic literature on physical fighting among adolescents in middle- and low-income countries.

## 2. Methods

### 2.1. Sample

In this study, the 2009 Pakistan Global School-based Health Survey (GSHS) data were used for secondary analysis. The GSHS-Pakistan was developed by the World Health Organization in collaboration with the United States Centers for Disease Control and Prevention, and the Ministry of Health, Pakistan. Detailed information on the data collection methods, questionnaire, procedures, and data may be found elsewhere (http://www.cdc.gov/gshs/). Briefly, a two-stage cluster sampling design was used to facilitate the collection of data representing all students in grade 8 or 9 in Pakistan. At Stage 1, schools were selected with a probability proportional to their enrollment size. At Stage 2, classrooms within the selected schools were randomly selected and all students in selected classes were eligible to participate. A total of 5192 students aged 11–16 years participated in the Pakistan contribution to the GSHS. This methodology has been validated elsewhere [28]. Any respondent below 11 years or above 16 years was coded as 11 and 16 years old, respectively.

The school response rate was 88% and the student response rate was 87%. The overall response rate was 76%. In a global comparison of 34 countries using the same methodology and essentially the same questionnaire, the average response rate was calculated at 84.14 (SD 8.39) based on the rates presented in the study. Participation in the survey was voluntary and all students were informed of the anonymous nature of the questionnaire. Answers were self-reported on a questionnaire with computer scannable answer sheet. With the exception of verifying heights and weights, no validation measures were used for the other responses in the survey, including the responses to items used for the present study. In the present study, 15 participants who did not have complete information on age (seven cases) and sex (eight cases) were excluded, which resulted in a final sample of 5177 (24.8% female).

### 2.2. Dependent Variable

Physical fighting as dependent variable was derived from one question in the GSHS: “During the past 12 months, how many times were you in a physical fight?” Response options ranged from “0 times”, “1 time”, “2 or 3 times”, “4 or 5 times”, “6 or 7 times”, “8 or 9 times”, “10 or 11 times” or “12 or more times”. For the purpose of our analyses, participants were classified as having participated in a physical fight if they reported being in two or more fights (N = 1107). If none or one fight was reported, participants were classified as not participating in a physical fight (N = 4053); for 17 records, this information was missing.

Studies on rough and tumble play and aggression have shown that there is a difference between aggression and roughness among adolescents. Roughness during play may be associated with aspects of peer affiliation, although victims may need to defend themselves [29]. Rough play is when a fight occurs without demanding submissiveness and distress to the victim [29]. On the other hand, aggressiveness goes beyond roughness when the aggressor is likely to persist until they achieve a level of submissiveness from the victim [30]. In this regard, the intention is achieve dominance over peers and is likely to target potential victims who appear weaker in order to defeat them, and achieve a higher dominance reflected by the number of victories [30]. Engagement in roughness and escalation to aggression with other peers was done to demonstrate dominance by taking on various fights [30].

In relation to the present study, the justification for cut-off in fights occurring at least two times or more was to include adolescents who may have been involved in aggressive behavior. Thus, the problematic behavior is when one person is considered to have been involved in fights several times, either as the aggressor or the victim. The the non-problematic behavior was considered to have occurred when one was not involved in fights at all or only once during the 12 months preceding the survey. This is because participation in one fight may not have been with the intention to achieve dominance but rather the fight could have been a random occurrence. The challenge is that data on the reason for participating in the fights is not available and setting the cut-off at this level was to distinguish between frequent fighting and no or non-frequent fighting.

### 2.3. Independent Variables

Five independent variables were investigated at the individual level (anxiety, suicide planning, truancy, physical activity, and bullying victimization) and four independent variables at the social level (presence of supportive parental figures, presence of helpful peers, extent of social network, and food insecurity). Details on how these variables were created are provided in Table 1.

### 2.4. Statistical Analysis

The distribution of selected independent variables within the dichotomized physical fighting variable was examined first. Differences between physical fighting involvement among the variables were screened for statistical significance using Rao–Scott chi-square test, which is a design-adjusted version of Pearson’s chi-square test for categorical variables, and the design-adjusted version of *t*-test for continuous variables (age). Two survey binary logistic regression models were created. These were intended to model the ability of the selected independent variables to predict the dichotomized physical fighting variable. The first model included all variables which were significant at the bivariate level. A second model adjusted only for age and sex. The measures of association were reported as adjusted (aOR) and unadjusted (OR) odds ratios and associated 95% confidence intervals (CI). All analyses were carried out using Stata 15 [31]. All proportions—expressed in percentages—are weighted, unless specified otherwise. Statistical significance was determined using *p*-value of <0.05, and 95% as well as 99% confidence intervals were calculated.

## 3. Results

Within the recall period, 19.3% (unweighted count: 1107) of participants reported being involved in two or more physical fights, most of whom were males (79.9%). The mean age of the sample was 14.2 years old (SD: 0.87). Having parents/guardians who understood the respondent’s problems and worries which was recorded as either most of the time or always was reported by 39.6%. Always having helpful peers was reported by 23.5% of respondents. Fifteen percent of respondents reported being bullied, and 12.2% reporting feeling lonely. Overall, 18.5% reported being physically active for at least 60 min per day, in the past seven days, for three or less days.

Table 2 shows the weighted distribution of selected factors according to physical fighting category. The bivariate analyses show that within all but three of the selected variables, significant differences existed between participants who had been involved in physical fights and those who were not. No statistically significant differences existed with regard to age, physical activity and food insecurity with physical fighting. Table 3 shows the distribution of variables by sex.

The age and sex adjusted analysis (Table 4) for all the variables found to be statistically significant in bivariate analysis, revealed statistically significant associations for all selected variables with the exception of age, supportive parental figures, and having no friends, compared to having three or more friends. However, using 99% confidence intervals, i.e., using significance level of <0.01, helpful peers and having two close friends compared to three or more close friends were also not statistically significantly associated with physical fight.

Table 5 shows the results from the final model that adjusted for all associated covariates that were found to be statistically significant—using *p*-value of <0.05—in the bivariate analysis; compared to those who did not report being involved in physical fighting, those who had been involved in physical fights were more likely to be males (OR 2.78; CI 2.04–3.78), have made suicide plan (OR 1.75; CI 1.31–2.33), have felt lonely (OR 1.44; CI 1.09–1.89), be truant (OR 1.63; CI 1.26–2.12), and have been bullied (OR 3.14; CI 2.32–4.25). Having no (OR 0.65; CI 0.44–0.95), one (OR 0.60; CI 0.47–0.78), or two (OR 0.81; CI 0.66–0.98) close friends, as opposed to having three or more close friends, had a protective effect against involvement in physical fights. The goodness-of-fit test revealed that this was a good multivariate logistic model for physical fight in Pakistani students (F: 2.05, *p*-value: 0.1103). However, using 99% CIs, loneliness, and having no or two close friends compared to three or more close friends were also not statistically significantly associated with reported physical fighting behavior.

## 4. Discussion

In this study, approximately 20% of the participants (Males = 79.9%) reported being involved in a physical fight within the recall period. This is within the range reported in other studies conducted in Asia between 2001 and 2013, where the prevalence of fights ranged from 14% to 38% [8,9,10,11], but relatively lower than reported in some studies from other regions in Sub-Saharan Africa [2,4,20], Europe and the United States [13,20].

Other studied risk factors for physical fighting such as male gender, bullying victimization, truancy, and depressive symptoms were found to have associations in this study conducted in Pakistan. Although the trend for male gender was found in this study as reported in other studies [4,8,11,15], it has been reported that more females are engaging in physical fights and violence [6,13,14] and no associations by gender have been found in another study [2]. Bullying victimization has been associated with poor mental health, truancy, and hunger [32]. This study found an association between physical fights and bullying victimization and truancy, as in other studies [2,4,33].

Loneliness was also associated with physical fights, which was observed in another study [8]. Availability of a suicide plan was also associated with physical fights, as observed in other studies [8,34]. Depressive symptoms such as sadness and loneliness are indicators of poor mental health that have been associated with physical fights and violence [13,32].

Factors that have been observed to be protective against physical fights have included parental guidance [2,4,14], having helpful peers [4], a positive school environment [2], the pressure of studies [33], and increased absolute wealth [3]. In this study, only having a few (0–2) close friends was found to be protective, which is consistent with results from a similar study among adolescents in Egypt that showed that only one close friend as opposed to none or more friends was protective [4]. Additionally, no protective association was observed between physical fights and parental support or guidance, which is similar to a study conducted in Indonesia [15]. In addition, no association was found for sedentary behavior in this study, although it was found in other studies [4,16].

One possible explanation for having few close friends being a protective factor as opposed to having helpful peers may be the positive influence that close, healthy relationships with peers have on the development of an adolescent. Whereas experiencing helpful peers may be a fleeting, superficial interaction, regularly socializing with a few close friends is more meaningful and has a deeper, lasting impact on the development of an adolescent, which includes their propensity to engage in deviant behaviors. The extant literature on adolescent development details the strong influence peers have on areas of adolescent development such as self-concept, academic aptitude, and deviant behavior [25,35,36,37,38].

The result of parental support or guidance offering no protective effect on adolescents engaging in physical fighting is an unusual finding. It contradicts a large body of empirical evidence, which has found that parental support and guidance exerts a protective effect on adolescents’ likelihood to participate in physical fighting [35,36,39]. However, the bulk of this research has been conducted in Western countries. Research specific to Arab countries suggests that parental support or warmth may not be as important to the mental health of adolescents; parenting style tends to be more authoritative in Arab countries, but this parenting style does not have adverse consequences on the mental health of adolescents as it does in Western countries [40,41,42]. This could be one explanation for why the present study of physical fighting among Pakistani adolescents did not find any protective effects for parental warmth and support.

### Strengths and Limitations

Several aspects of the present study lend itself to the reliability of the results. The sample was nationally representative of all school-attending adolescents aged 11–16 years in Pakistan. The sample size was large and the sampling procedure robust to allow for valid conclusions to be made from the performed analyses. The analyses presented here also take into account the clustered multi-stage sampling design. The survey questionnaire piloted extensively cross-nationally with cross-cultural settings in mind. All surveys were carried out in an environment which allowed for anonymous response to guard confidentiality.

However, the results must still be examined in light of several limitations. The cross-sectional nature of the data does not allow for an examination of causality. The data are silent on adolescents who were either not present at the time of the survey or those who may not attend school at all. This latter category of students are potentially the most at risk for being involved in physical violence. While all the data which were utilized for this study were self-reported, even in the context of an anonymous survey, responses are subject to social-desirability bias. Despite cross-national and cross-cultural validation of the survey tool, some questions may have been interpreted differently from their original intent by the students. For example, the question “How many close friends do you have?” could be interpreted differently depending on individual and cultural differences in qualities a person should exhibit to be considered a friend rather than an acquaintance. It is also important to note that the limited age of the study fails to take into consideration a more expanded understanding of behaviors, which may have been influenced by events occurring prior to adolescence. By the same notion, the limited age range also is not able to capture how these behaviors manifest long after adolescence.

## 5. Conclusions

The present study adds to the growing body of epidemiologic literature on physical fighting among adolescents in middle- and low-income countries. Our findings suggest that risk factors for physical fighting compiled from research conducted on adolescents in high-income countries may be generalizable to the adolescents living in middle- and low-income countries, and specifically to adolescents in South Asia. Interestingly, the protective factors delineated from research in high-income countries may not be applicable to this specific population. The protective factors of parental support and helpful peers were not found to have such a protective effect in our sample of Pakistani adolescents. It may be advisable that public health interventions designed to prevent physical fighting in Pakistan and similar countries should not leverage parents and peers in their efforts, but rather focus on involving close friends or helping those without close friends to develop these relationships, which were found to have a protective effect against physical fighting.

## Figures and Tables

**Table 1:** Independent variable derivation from GSHS-Pakistan survey data (2009).

Survey Question	Coding	Variable
**Individual level variables**		
How old are you	11–17 years (coded categorically)	Age
What is your sex?	Male (1); Female (0)	Sex
During the past 12 months, how often have you been so worried about something that you could not sleep at night?	Most of the time/always (1); Never/rarely/sometimes (0)	Anxiety
During the past 12 months, did you make a plan about how you would attempt suicide?	Yes (1); No (0)	Suicide Plan
During the past 12 months, how often have you felt lonely?	Most of the time/always (1); Never/rarely/sometimes (0)	Loneliness
During the past 30 days, how many days did you miss classes or school without permission?	0–2 times (0); 3 to or more days (1)	Truancy
During the past 30 days, on how many days	0 times (0); 1 or more times (1)	Bullying victimization
During the past 7 days, on how many days were you physically active for a total of at least 60 min per day?	3 days or less (0); 4 days or more (1)	Physical activity
How much time do you spend during a typical or usual day sitting and watching television, playing computer games, talking with friends, or doing other sitting activities?	2 h or less (0); 3 h or more (1)	Sedentary
During the past 12 months, how many times were you in a physical fight?	0–1 times (0) 2 or more times (1)	In a fight
**Social level variables**		
During the past 30 days, how often did your parents or guardians understand your problems and worries?	Most of the time/always (1); Never/rarely/sometimes (0)	Supportive parental figures
During the past 30 days, how often were most of the students in your school kind and helpful?	Always (1); Most of the time/Never/rarely/sometimes (0)	Helpful peers
How many close friends do you have?	0 close friends (0); 1 close friends (1); 2 close friends (2); 3+ close friends (3)	Close friends
During the past 30 days, how often did you go hungry because there was not enough food in your home?	Most of the time/always (1); Never/rarely/sometimes (0)	Food insecurity

**Table 2:** Distribution of selected factors according to categories of physical fighting among school-attending adolescents in Pakistan (2009).

Survey Question	Coding	Variable		
Variable	Physical Fight = no (n = 4053 *)	Physical Fight = yes (n = 1107 *)	*p*-Value	Chi-Square F/t-Value
Age-mean (SD)	14.1 (0.85)	14.2 (0.93)	0.242	1.20
Sex (Male)	56.6	79.9	<0.001	36.395
Anxiety	7.4	12.6	0.001	13.271
Suicide Plan	6.2	12.8	<0.001	50.099
Loneliness	10.9	17.6	<0.001	17.260
Truancy	5.1	10.4	<0.001	25.898
Bullying Victimization	10.8	32.5	<0.001	86.111
Physical activity	17.3	23.3	0.060	3.917
Sedentary	7.0	13.0	<0.000	26.005
Supportive parental figures	41.1	33.2	0.020	6.271
Helpful peers	24.8	18.4	0.023	5.937
Social network				
0 close friends	8.2	7.1	<0.001	9.821
1 close friend	28.4	19.9		
2 close friends	23.9	22.9		
3+ close friends	39.5	50.2		
Food insecurity	5.8	5.6	0.849	0.037

All variables are expressed as proportions (in %) except for age (mean and standard deviation). * Unweighted.

**Table 3:** Distribution of selected variable categories by sex among a nationally representative sample of Pakistani adolescents (2009).

Variable	Male	Female	*p*-Value	Chi-Square F/t-Value
Age (mean)	14.2 (0.95)	14.1 (0.70)	0.102	1.70
Physical Fight	25.5	9.9	<0.001	36.395
Anxiety	8.1	8.9	0.667	0.190
Suicide Plan	7.6	7.4	0.900	0.016
Loneliness	11.5	13.3	0.329	0.997
Truancy	7.9	3.4	0.007	8.893
Bullying Victimization	18.0	10.1	0.667	3.657
Physical activity	20.9	14.6	0.245	1.425
Sedentary	8.9	6.9	0.235	1.490
Supportive parental figures	33.3	49.4	<0.001	14.740
Helpful peers	22.6	24.9	0.536	0.395
Social network				
0 close friends	7.1	9.5	0.001	8.007
1 close friend	24.0	31.1		
2 close friends	23.2	24.6		
3+ close friends	45.8	34.8		
Food insecurity	6.6	4.6	0.148	2.245

Percentages of the total within each category are listed.

**Table 4:** Multivariate analysis of physical fighting among school-attending adolescents in Pakistan (2009).

Variable	OR	95% CI (99% CI)	*p*-Value
Age	1.11	0.93–1.33 (0.87–1.42)	0.233
Sex	3.05	2.05–4.53 (1.78–5.23)	<0.001
Anxiety	1.87	1.37–2.56 (1.23–2.86)	<0.001
Suicide Plan	2.28	1.79–2.90 (1.65–3.16)	<0.001
Loneliness	1.85	1.38–2.48 (1.24–2.75)	<0.001
Truancy	1.87	1.41–2.48 (1.27–2.74)	<0.001
Bullying Victimization	3.65	2.73–4.88 (2.46–5.42)	<0.001
Sedentary	1.91	1.35–2.71 (1.20–3.07)	0.001
Supportive parental figures	0.82	0.65–1.04 (0.60–1.13)	0.97
Helpful peers	0.69	0.50–0.97 (0.44–1.09)	0.034
Social network			
0 close friends	0.77	0.51–1.16 (0.44–1.35)	0.204
1 close friends	0.62	0.50–0.77 (0.46–0.83)	<0.001
2 close friends	0.81	0.66–0.98 (0.62–1.06)	0.036
3+ close friends	–	–	–

OR, Odds Ratio; 95% and 99% CI, 95% and 99% Confidence Intervals. All estimates are adjusted for age and sex; age; or sex.

**Table 5:** Multivariate analysis of physical fighting among school-attending adolescents in Pakistan (2009).

Variable	OR	95% CI (99% CI)	*p*-Value
Sex	2.78	2.04–3.78 (1.83–4.23)	<0.001
Anxiety	1.36	0.95–1.94 (0.84–2.20)	0.087
Suicide Plan	1.75	1.31–2.33 (1.19–2.58)	0.001
Loneliness	1.44	1.09–1.89 (0.99–2.08)	0.011
Truancy	1.63	1.26–2.12 (1.15–2.33)	0.001
Bullying Victimization	3.14	2.32–4.25 (2.08–4.74)	<0.001
Sedentary	1.44	0.97–2.14 (0.84–2.46)	0.067
Supportive parental figures	0.91	0.71–1.18 (0.64–1.30)	0.467
Helpful peers	0.71	0.50–1.01 (0.44–1.140)	0.054
Social network			
0 close friends	0.65	0.44–0.95 (0.38–1.10)	0.029
1 close friends	0.60	0.47–0.78 (0.43–0.85)	<0.001
2 close friends	0.81	0.66–0.98 (0.62–1.05)	0.034
3+ close friends	–	–	–

OR, Odds Ratio; 95% and 99% CI, 95% and 99% Confidence Intervals. All estimates are adjusted for all variables listed in the table.
